# Information provision and retrieval by registered salespersons from consumers during over-the-counter drug sales – a questionnaire survey

**DOI:** 10.1186/s12913-021-07343-x

**Published:** 2021-12-13

**Authors:** Hayato Kizaki, Misato Mochizuki, Yutaka Yoshida, Kaori Ishikawa, Miya Ohishi, Hiroki Satoh, Yasufumi Sawada, Satoko Hori

**Affiliations:** 1 Division of Drug Informatics, Keio University Faculty of Pharmacy, 1-5-30 Shibakouen, Minato-ku, Tokyo, 105-8512 Japan; 2 Faculty of Pharmaceutical Sciences, The University of Tokyo, 7-3-1 Hongo, Bunkyo-ku, Tokyo, 113-0033 Japan; 3 Ain Pharmaciez inc., 2-4-30Higashisapporo, Shiraishi-ku, Sapporo, Hokkaido 003-0005 Japan; 4 Graduate School of Pharmaceutical Sciences, The University of Tokyo, 7-3-1 Hongo, Bunkyo-ku, Tokyo, 113-0033 Japan

**Keywords:** Self-medication, Over-the-counter drug, Registered salespersons, Pharmacies, Pharmacists

## Abstract

**Background:**

In Japan, non-pharmacists who are accredited as registered salespersons can sell over-the-counter (OTC) drugs, and they play a very important role in supporting proper OTC drug use by consumers. The purpose of this study was to evaluate information provided to and information collected from consumers, and cooperation with pharmacists during OTC drug sales by registered salespersons, and to clarify their related concerns and behaviors.

**Methods:**

A cross-sectional questionnaire-based survey of 385 registered salespersons working at 56 drugstores throughout Japan was conducted. Based on the questionnaire survey, the frequency of information provision/collection in various categories was determined for the registered salespersons. The relation between concerns of registered salespersons relating to OTC drug sales and the frequency of information provision/collection was examined. The frequency of consultation of registered salespersons with a pharmacist was calculated for registered salespersons with/without in-store pharmacists. The χ-square test or Fisher’s exact test was performed to assess the significance of differences.

**Results:**

Two hundred and seven registered salespersons (53.7%) responded completely. A greater number of OTC drug purchasers per day was associated with a greater frequency of information provision about “side effects” and information collection about “favorite items” (alcohol, tobacco, health foods, etc.) (*p* < 0.05). One hundred and thirty-nine (67.2%) participants had concerns about “interactions between OTC drugs and prescription drugs”, and these concerns were related to the frequency of information provision/collection (*p* < 0.05). Regarding the frequency of consultation with a pharmacist, 35 of 46 participants (76.1%) working with pharmacists answered “always” or “usually”, whereas only 19 of 161 participants (11.8%) working without full-time pharmacists answered “always” or “usually”. More than half of the registered salespersons thought that cooperation with a pharmacist was necessary when they were “asked about concomitant use with prescription drugs” or “told that side effects happened.”

**Conclusions:**

The results of this study show that experienced registered salespersons selling OTC drugs are more likely to collect information from consumers and to provide information to consumers. It appears to be important for registered salespersons to cooperate with pharmacists in order to provide and collect appropriate information about concomitant medications.

**Supplementary Information:**

The online version contains supplementary material available at 10.1186/s12913-021-07343-x.

## Background

The core of self-medication is to take responsibility for one’s own health and to take care of mild medical conditions by using over-the-counter (OTC) drugs that can be purchased and used without a prescription [1]. However, there can be interactions between OTC drugs and prescription drugs, health foods, or supplements, as well as side effects and misuse of OTC drugs [2, 3]. In fact, problems related to improper use, such as not reading package inserts, not complying with the recommended dosage, and using prescription drugs and OTC drugs simultaneously without expert advice, occur worldwide [4–7]. Also in a survey conducted in Hiroshima, Japan, in 2009, it was reported that 34.8% of the 508 participants had taken OTC drugs in combination with prescription drugs [8]. Thus, medical professionals should encourage the proper purchase and use of OTC drugs by consumers.

In some countries, it is possible for authorized persons to sell OTC drugs without being pharmacists. In Nigeria, proprietary and patent medicine vendors (with no formal training in pharmacy) provide a variety of health services, including OTC drug sales [9, 10]. In Bangladesh, drug shops can sell medicines by obtaining approval after a 12-week training course [11]. In Japan, “registered salespersons” who have passed examinations conducted by prefectural governments, regardless of their educational background or work experience, are mainly responsible for the OTC drug sales in drugstores.

The system of registered salespersons in Japan was launched on June 14, 2006, when an amendment of the Pharmaceutical Affairs Law was promulgated to revise the entire OTC drug sales system. In Japan, consumers can purchase OTC drugs at drug stores (frequently selling many other items such as food, drinks, cleaning products, and so on) and pharmacies without prescription, and registered salespersons are permitted to sell low-risk OTC drugs. OTC drugs were categorized into 3 classes according to the degree of risk. Moreover, a new profession of “registered salespersons”, who are permitted to sell low-risk OTC drugs, was established. Registered salespersons can sell Class 2 (moderate risk) and Class 3 (relatively low risk) drugs, but not Class 1 (relatively high risk) drugs. For example, Class 2 drugs include pseudoephedrine. Some of the OTC drugs that can be sold by registered salespersons in Japan must not be used by patients with certain diseases such as diabetes. Thus, it is important for registered salespersons to be familiar with the content of drug package inserts, and also to collect information from consumers and provide information to consumers before selling some OTC drugs.

It has been more than 10 years since the system of registered salespersons was adopted in Japan. However, there has been insufficient research on the status of information provision and information collection, as well as the concerns and anxieties faced by registered salespersons during OTC drug sales. It has recently been reported that in the case of OTC drug sales to patients with chronic kidney disease (CKD), about half of the registered salespersons have on occasion sold inappropriate OTC drugs to CKD patients [12], and thus it appears that the proper sale of OTC drugs by registered salespersons is still difficult. Since the awareness and knowledge of the registered salespersons were improved by the intervention of pharmacists [12], cooperation between registered salespersons and pharmacists in drugstores is considered to be important.

## Aim of this study

The present study aimed to clarify the status of information provided to and information collected from consumers during OTC drug sales by registered salespersons, as well as the actual status of cooperation between registered salespersons and pharmacists, and factors related to the practice of these behaviors, by means of a questionnaire survey.

## Methods

### Design and participants

The study design was a cross-sectional questionnaire-based survey of 385 registered salespersons working at 56 drugstores throughout Japan. These were all drugstores operated by one company, Ain Pharmaciez Inc., and all the participating registered salespersons worked in them. In Japan, registered salespersons mainly work in drugstores, and some work in pharmacies. Pharmacists are permitted to sell all types of OTC drugs, but registered salespersons are permitted to sell only low-risk OTC drugs.

We developed original questionnaires about information collection and information provision regarding the proper use of drugs by registered salespersons. Questionnaires and a document explaining the research were sent to the registered salespersons by management staff of the company in October 2017 via post. The document stated that participants in the survey would remain anonymous. Subjects who read the document and agreed to cooperate in this study were asked to complete the questionnaire survey on paper. The completed questionnaires were returned directly by individual participants to the Faculty of Pharmacy, the University of Tokyo, via post. The responses were not disclosed to the company. Questionnaires with incomplete answers (missing answers, double answers, etc.) were excluded from the analysis.

### Questionnaire survey

The questionnaire survey was conducted in Japanese. The questionnaire used multiple-choice and descriptive questions. The questions asking about the frequency of information provision and information collection were prepared based on a previous study, in which a questionnaire was developed to survey social needs for registered salespersons [13]. The questions asking about concerns that registered salespersons have in relation to daily OTC drug sales and opinions about problems or challenges with OTC drug sales were based on the input of 24 registered salespersons who participated in a workshop-type training session for registered salespersons. These 24 registered salespersons were also included in the study population. In order to ensure the validity of the questionnaire, a person with practical experience as a registered salesperson (Y. Yoshida) reviewed the survey questions. Reliability analysis, such as calculating Cronbach alpha coefficients, was not conducted, because this study was not designed to develop a scale or to evaluate a concept.

The questionnaire contained 33 items about the following topics: (1) basic information about the registered salesperson; (2) frequency of information collection / information provision contributing to promotion of the proper use of drugs by the registered salesperson; (3) concerns that the registered salesperson has in relation to daily OTC drug sales; (4) cooperation with the pharmacist, etc.

1. Basic information about the registered salesperson

Details of age of the registered salesperson, gender, years of experience, and the number of consumers per day for OTC drug sales were requested.

(2) Frequency of information collection / information provision by the registered salesperson

Frequency of information provision about “brand name”, “indications”, “dosage”, “side effects”, “ingredients”, “precautions for use”, and “precautions for storage and handling” was scored on a four-point scale: “1; always”, “2; sometimes”, “3; not often, “4; not at all”. The reason why they do (can) not provide information about “precautions for use” was asked in the multiple-choice question only for participants who answered “not often” or “not at all”. Frequency of information collection about “symptoms”, “age”, “medical history”, “pregnancy (for consumers of relevant age and sex)”, “concomitant medications”, “favorite items (alcohol, tobacco, health foods, etc.)”, and “allergies” was also scored on a similar four-point scale. The reason why they do (can) not collect information about “concomitant medications” was asked in the multiple-choice question only for participants who answered “not often” or “not at all”.

(3) Concerns that the registered salesperson has in relation to daily OTC drug sales

Concerns of the registered sales person regarding drug sales were scored on a five-point scale:“1; not at all”, “2; not very much”, “3; neither”, “4; somewhat worried”, “5; very worried”.

(4) Cooperation with the pharmacist

Frequency of consultation with a pharmacist was scored on a five-point scale: “1; not at all”, “2; not often”, “3; neither”, “4; usually”, “5; always”.

### Analysis

We examined whether gender (male/female), age group (10’s / 20’s / 30’s / 40’s / 50’s / 60 or more), years of experience as a registered sales person (less than 1 year / 1–3 years / 3–5 years / 5 years or more) and number of OTC customers served per day (1–10 persons / 10–20 persons / 20–30 persons / 30 persons or more) by the registered sales person were related to the frequency of information provision and information collection. For each question related to information provision and information collection, the proportion of respondents who answered “always” or “sometimes” was calculated and compared according to gender, age group, years of experience as a registered sales person, and number of OTC customers served per day.

In order to examine the relationship between the frequency of information provision or information collection and the concerns of registered salespersons, participants were divided into 2 groups. According to the frequency of appropriate information provision and information collection, a high frequency (HF) group and a low frequency (LF) group were defined. The high frequency group included participants who replied “always” or “sometimes” to all 7 items; information provision on “indications”, “dosage”, “side effects” and “precautions for use”, information collection on “medical history”, “pregnancy”, “presence of concomitant medications”, which are very important for proper use. The low frequency group included all other participants. The proportions of participants in the high and low frequency groups who responded “very worried” or “somewhat worried” to questions on the registered salespersons’ concerns regarding drug sales were compared.

We also analyzed whether the registered salespersons’ needs for cooperation with pharmacists differ depending on whether they were currently working with or without a full-time pharmacist at the drug store. The differences in the situations where registered salespersons felt that they needed cooperation with a pharmacist were compared between groups with and without full-time pharmacists.

In the test of differences between groups, the χ-square test was performed at the 5% level of significance. When 20% or more of the expected frequencies were less than 5, Fisher’s exact test was performed. IBM SPSS ver.25 (IBM, USA) was used for Fisher’s exact test and Microsoft Excel ver.16.28 was used for all other analyses.

## Results

### Background of the participants

The questionnaires were sent to 385 registered salespersons. Two hundred and sixty-one (67.8%) registered salespersons responded, of which 207 (53.7%) provided complete and acceptable questionnaires. The background of these participants is described in Table 1. About the half of the participants had less than 3 years of experience as registered salespersons for OTC drugs. In this study, 46 (22.2%) of the participants replied that a full-time pharmacist was present in the store where they worked.

**Table 1:** Backgrounds of the subjects (registered salespersons)

*N* = 207	n(%)
Gender	
Male	59(28.5)
Female	148(71.5)
Age	
10’s	0 (0.0)
20’s	108(52.1)
30’s	60(29.0)
40’s	26(12.6)
50’s	8 (3.9)
60 or more	5 (2.4)
Years of experience as a registered sales person	
Less than 1 year	32(15.5)
1–3 years	71(34.3)
3–5 years	29(14.0)
5 years or more	75(36.2)
Average number of consumers per day for OTC drug sales	
1–10 persons	128(61.8)
10–20 persons	42(20.3)
20–30 persons	12 (5.8)
30 persons or more	12 (5.8)
Don’t sell now	13 (6.3)
Whether or not there is a full-time pharmacist in the store where the registered sales person works	
Yes	46(22.2)
No	161(77.8)

### Information provision and information collection by registered salespersons contributing to the promotion of proper use of drugs

The frequencies of information provision by registered salespersons and information collection from consumers are summarized in Table 2. Regarding “precautions for use”, 62 (30.0%) participants answered “not often” or “not at all”. Reasons for these responses were “there is no time”, “the precautions are given in the package insert” or “consumers themselves would understand precautions” etc. Regarding “presence of concomitant medications”, 24 (11.5%) participants answered “not often” or “not at all”. Reasons for these responses were “there is no time”, “hesitation to confirm” or “I’ve never had a problem without confirming”, etc.

**Table 2:** Frequencies of information provision to consumers and information collection from consumers

N = 207	Always	Sometimes	Not often	Not at all
Information provision				
Brand name	91 (44.0)	83 (40.1)	28 (13.5)	5 (2.4)
Indications	151 (72.9)	49 (23.7)	6 (2.9)	1 (0.5)
Dosage	94 (45.4)	99 (47.8)	13 (6.3)	1 (0.5)
Side effects	42 (20.3)	101 (48.8)	59 (28.5)	5 (2.4)
Ingredients	71 (34.3)	116 (56.0)	18 (8.7)	2 (1.0)
Precautions for use	45 (21.7)	100 (48.3)	53 (25.6)	9 (4.3)
Precautions for storage and handling	14 (6.8)	68 (32.9)	100 (48.3)	25 (12.1)
Information collection				
Symptoms	191 (92.3)	14 (6.8)	1 (0.5)	1 (0.5)
Age	63 (30.4)	107 (51.7)	33 (15.9)	4 (1.9)
Medical history	46 (22.2)	109 (52.7)	45 (21.7)	7 (3.4)
Pregnancy (for consumers of relevant age and sex)	54 (26.1)	97 (46.9)	50 (24.2)	6 (2.9)
Concomitant medications	82 (39.6)	101 (48.8)	21 (10.1)	3 (1.4)
Favorite items†	13 (6.3)	68 (32.9)	102 (49.3)	24 (11.6)
Allergies	54 (26.1)	103 (49.8)	44 (21.3)	6 (2.9)

Numbers in parentheses indicate percentage of the total of 207 respondents

†Alcohol, tobacco, health foods, etc.

The relationship between the frequency of information provision/collection and the number of consumers served per day with OTC drugs by the registered salespersons is shown in Table 3. The percentages of participants who answered “always” or “sometimes” about “side effects” were 66.4% (1–10), 71.4% (10–20), 100% (20–30), and 91.7% (30 or more). A significant difference was found between these groups (Fisher’s exact test, *p* < 0.05). Moreover, the percentages of participants who answered “always” or “sometimes” about “favorite items” were 35.2% (1–10), 35.7% (10–20), 75.0% (20–30), and 66.7% (30 or more). A significant difference was found between these groups (Fisher’s exact test, *p* < 0.05). The relationship between the frequency of information provision/collection and gender/age group/years of sales experience is shown in Supplementary Tables 1–3.

**Table 3:** Frequency of information provision and information collection by number of consumers per day for OTC drug sales by registered salespersons

	1–10 persons (*n* = 128)	10–20 persons (*n* = 42)	20–30 persons (n = 12)	30 or more (n = 12)
Information provision				
Brand name	107 (83.6)	38 (90.5)	11 (91.7)	10 (83.3)
Indications	125 (97.7)	41 (97.6)	12 (100)	12 (100)
Dosage	119 (93.0)	42 (100)	12 (100)	12 (100)
Side effects *	85 (66.4)	30 (71.4)	12 (100)	11 (91.7)
Ingredients	116 (90.6)	40 (95.2)	12 (100)	12 (100)
Precautions for use	88 (68.8)	31 (73.8)	11 (91.7)	10 (83.3)
Precautions for storage and handling	49 (38.3)	15 (35.7)	9 (75.0)	7 (58.3)
Information collection				
Symptoms	128 (100)	42 (100)	12 (100)	12 (100)
Age	105 (82.0)	35 (83.3)	11 (91.7)	11 (91.7)
Medical history	91 (71.1)	36 (85.7)	11 (91.7)	11 (91.7)
Pregnancy (for consumers of relevant age and sex)	90 (70.3)	33 (78.6)	10 (83.3)	10 (83.3)
Concomitant medications	112 (87.5)	38 (90.5)	12 (100)	12 (100)
Favorite items† *	45 (35.2)	15 (35.7)	9 (75.0)	8 (66.7)
Allergies	97 (75.8)	33 (78.6)	10 (83.3)	11 (91.7)

The number of registered salespersons who answered “always” or “sometimes” was counted according to the number of sales per day. Subjects who answered “don’t sell now” were excluded from the table. Numbers in () indicate percentage of all respondents

*p < 0.05

† Alcohol, tobacco, health foods, etc.

### Relationship between information provision/collection by registered salespersons and their concerns

The largest number of participants (139, 67.2%) answered that they were “very worried” or “somewhat worried” about being “unable to answer questions about concomitant use with prescription drugs” (Table 4).

**Table 4:** Concerns of registered salespersons about drug sales

N = 207	Not at all	Not very much	Neither	Somewhat worried	Very worried
Unable to judge whether to recommend medical consultation	22 (10.6)	83 (40.1)	72 (34.8)	28 (13.5)	2 (1.0)
Unable to answer consumer’s questions about OTC drugs	18 (8.7)	74 (35.7)	55 (26.6)	49 (23.7)	11 (5.3)
Unable to explain well about the indications	20 (9.7)	87 (42.0)	52 (25.1)	40 (19.3)	8 (3.9)
Unable to explain well about the dosage	52 (25.1)	91 (44.0)	36 (17.4)	22 (10.6)	6 (2.9)
Unable to respond when a consumer says that side effects have occurred	12 (5.8)	34 (16.4)	61 (29.5)	67 (32.4)	33 (15.9)
Unable to answer questions about concomitant use with prescription drugs	16 (7.7)	19 (9.2)	33 (15.9)	77 (37.2)	62 (30.0)
Unable to answer questions about concomitant use with healthy foods	12 (5.8)	40 (19.3)	63 (30.4)	73 (35.3)	19 (9.2)
Unable to judge whether pregnant or breast-feeding women can use OTC drugs	14 (6.8)	54 (26.1)	54 (26.1)	68 (32.9)	17 (8.2)
Unable to judge whether infants or children can use OTC drugs	17 (8.2)	67 (32.4)	59 (28.5)	50 (24.2)	14 (6.8)
Unable to judge whether the elderly can use OTC drugs	16 (7.7)	51 (24.6)	52 (25.1)	70 (33.8)	18 (8.7)
Unable to explain well to foreigners about OTC drugs	14 (6.8)	24 (11.6)	32 (15.5)	58 (28.0)	79 (38.2)
Absence of pharmacist or other registered sales person who can resolve doubts	74 (35.7)	73 (35.3)	36 (17.4)	18 (8.7)	6 (2.9)
Unable to speak to consumers actively	80 (38.6)	76 (36.7)	39 (18.8)	9 (4.3)	3 (1.4)
Advice rejected by consumers	62 (30.0)	90 (43.5)	39 (18.8)	14 (6.8)	2 (1.0)
Unable to have confidence in his or her explanation	26 (12.6)	57 (27.5)	64 (30.9)	45 (21.7)	15 (7.2)
Unable to make consumers understand	29 (14.0)	90 (43.5)	76 (36.7)	11 (5.3)	1 (0.5)

Numbers in parentheses indicate percentage of the total of 207 respondents

We examined whether concerns in OTC drug sales differ depending on the frequency of information provision and information collection (HF group; *N* = 92, LF group; *N* = 115) (Table 5). The percentage of participants who worried about being “unable to answer questions from consumers about OTC drugs”, “unable to answer questions about concomitant use with prescription drugs”, “unable to answer questions about concomitant use with health foods”, or “unable to have confidence in my explanation” was significantly higher in the LF group (χ-square test, *p* < 0.05).

**Table 5:** Percentage of registered salespersons that have concerns about drug sales relating to proper use of medication

	HF group(n = 92) (%)	LF group(n = 115) (%)
Unable to judge whether to recommend medical consultation	12.0	16.5
Unable to answer consumer’s questions about OTC drugs	20.7	35.7*
Unable to explain well about the indications	21.7	24.3
Unable to explain well about the dosage	12.0	14.8
Unable to respond when a consumer says that side effects have occurred	41.3	53.9
Unable to answer questions about concomitant use with prescription drugs	58.7	73.9*
Unable to answer questions about concomitant use with healthy foods	35.9	51.3*
Unable to judge whether pregnant or breast-feeding women can use OTC drugs	37.0	44.3
Unable to judge whether infants or children can use OTC drugs	28.3	33.0
Unable to judge whether the elderly can use OTC drugs	35.9	47.8
Unable to explain well to foreigners about OTC drugs	60.9	70.4
Absence of pharmacist or other registered sales person who can resolve doubts	9.8	13.0
Unable to speak to consumers actively	3.3	7.8
Advice rejected by consumers	5.4	9.6
Unable to have confidence in his or her explanation	19.6	36.5*
Unable to make consumers understand	5.4	6.1

The participants who answered “always” or “sometimes” to all 7 items (information provision on “indications”, “dosage”, “side effects” and “precautions for use”, information collection on “medical history”, “pregnancy”, “presence of concomitant medications”) that are highly important for proper use were included in the high frequency (HF) group. The others were included in the low frequency (LF) group

* *p* < 0.05

### Cooperation with pharmacists

Regarding the frequency of consultation with a pharmacist when worries or anxieties arise about drugs, 54 (26.1%) participants answered “always” or “usually” (Supplementary Table 4). In particular, 35 (76.1%) participants who work with pharmacists at their stores answered “always” or “usually”, whereas only 19 (11.8%) participants who work at stores without full-time pharmacists answered “always” or “usually”. There was a significant difference between the two groups (χ-square test, *p* < 0.05). Participants who work with pharmacists at their stores (working with pharmacist group) often needed cooperation with a pharmacist (Table 6). On the other hand, in situations such as “when asked about concomitant use with prescription drugs”, “when told that side effects happened” or “when it’s difficult to explain to foreign consumers about drugs”, the need for cooperation with pharmacists was similar whether or not there was a full-time pharmacist.

**Table 6:** Percentage of participants who need cooperation of pharmacists in various situations

N = 207	Working with pharmacist (*n* = 46) (%)	Not working with pharmacist (*n* = 161) (%)
When can’t judge whether to recommend medical consultation to the consumer	37.0	25.5
When asked about the effects of drugs	43.5	23.6*
When asked about the side effects of drugs	63.0	42.9*
When asked about the indication of drugs	34.8	16.1*
When asked about the dosage of drugs	19.6	6.8*
When told that side effects happened	73.9	50.3
When asked about concomitant use with prescription drugs	84.8	79.5
When asked about concomitant use with healthy foods	50.0	25.5*
When deciding whether or not OTC drugs could be sold to pregnant or breast-feeding women	58.7	37.9*
When deciding whether or not OTC drugs could be sold to infants or children	50.0	26.7*
When deciding whether or not OTC drugs could be sold to the elderly	56.5	32.9*
When it’s difficult to explain to foreign consumers about drugs	26.1	14.9

* p < 0.05

## Discussion

This study is the first detailed investigation of the working practices of registered salespersons, who are permitted to sell low-risk OTC drugs independently of pharmacists. Our questionnaire survey indicates that experienced registered salespersons were more likely to collect information from consumers and to provide information to consumers on proper use of OTC drugs. Concerns about “interactions between OTC drugs and prescription drugs” were related to the frequency of information provision and information collection. The need of registered salespersons for cooperation with pharmacists was generally high.

In Nigeria, there are proprietary and patent medicine vendors (PPMVs), which are similar to registered salespersons in Japan. In Nigeria, where medical resources are scarce, PPMVs can potentially provide access to essential drugs across a wide range of diseases, but it has been reported that the quality of their services is poor [10]. The services of registered salespersons in Japan are operated under strict regulations. Our findings suggest that the quality of services provided by registered salespersons in Japan is generally good, because information provision and collection by registered salespersons were performed with high frequency. On the other hand, about 30% participants answered “not often” or “not at all” regarding the frequency of information provision about “precautions for use” to consumers in this survey, and this may be insufficient. Some participants mentioned “the precautions are given in the package insert” or “consumers themselves would understand precautions” as the reason for not providing information to consumers. Moreover, about 12% participants answered “not often” or “not at all” regarding the frequency of information collection about “concomitant medications” from consumers. It has been reported that many consumers are not fully aware of the interactions between health foods and drugs [14], and thus communication between registered salespersons and consumers may be very important. Few consumers are willing to proactively consult with health care professionals [15], and many believe that sufficient information can be obtained from the package or package insert of the drug. Thus, registered salespersons in Japan should provide information more actively.

There was a relation between the number of consumers served per day and the frequency of information provision about “side effects” and information collection about “favorite items”. In other words, greater experience of OTC drug sales may make it easier to provide and collect such information.

Most participants (88.4%) answered they “always” or “sometimes” collect information on concomitant medications from consumers. Moreover, in the LF group, the percentage of registered salespersons who worried about concomitant use with prescription drugs or health foods was significantly higher. These results suggested that concerns regarding poor knowledge of drug-drug or drug-food interactions may inhibit registered salespersons from collecting and providing information about proper drug use. In Japan, registered salespersons are required to receive a minimum of 12 h of training per year. Therefore, establishing a new training program focusing on solving these concerns is likely to be helpful.

In Japan, OTC drugs are categorized into 3 classes according to the degree of risk, and registered salespersons can sell only low-risk OTC drugs. On the other hand, pharmacists can sell all types of OTC drugs. Previous research has found that pharmacists’ interventions may improve registered salespersons’ awareness and knowledge of OTC drugs [12], therefore it may be important for registered salespersons to cooperate with pharmacists. Indeed, about one-fourth of the registered salespersons in this study consulted with pharmacists when they felt uncertain or anxious about an OTC drug. In particular, three-fourths of registered salespersons who had full-time pharmacists working at their stores consulted with pharmacists. The proportion was significantly higher than that of consultation with pharmacists by registered salespersons working at stores without full-time pharmacists. Furthermore, in many situations, the proportion of registered salespersons who thought that “the cooperation of the pharmacist was necessary” was higher among registered salespersons who worked with pharmacists compared with those who did not. However, there was no significant difference in situations such as “when asked about concomitant use with prescription drugs” or “when told that side effects happened”, depending on the presence or absence of a full-time pharmacist. More than half of the respondents thought that cooperation with pharmacists was necessary when they were “asked about concomitant use with prescription drugs” or “told that side effects happened”. Therefore, in cases where detailed knowledge regarding drug interactions and adverse drug reactions is required, cooperation between registered salespersons and pharmacists may be required, regardless of whether or not a full-time pharmacist is present.

Nearly 70% (67.2%) of the participants were worried that they could not answer questions from consumers regarding the concomitant use of OTCs with prescription drugs. To determine whether OTC drugs can be used concomitantly with other drugs, especially prescription drugs, requires more advanced knowledge about drug properties than would be available to registered salespersons. Therefore, we need to develop a system that allows registered salespersons to easily consult a pharmacist or to share their role with a pharmacist when necessary.

There are several limitations in this study. The participants in this survey were registered salespersons who work at drugstore chains located throughout Japan, operated by a single corporation. We cannot rule out the possibility that different trends may be seen in other drugstores, especially in the case of small chains of drugstores that might have a regional bias of behavior. Moreover, because the questionnaire survey was self-administered, it is possible that there is a recall bias. Considering the possibility of social desirability bias, the frequency of information provision or information collection may be overestimated. This study is a cross-sectional study, and provides no information about causality.

The main strength of this study is that the current status of information provision and information collection was clarified from the perspective of registered salespersons regarding their contribution to self-medication. The findings of this study may provide basic information for improving the input of non-pharmacists and non-medical personnel into the OTC drug sales system.

## Conclusion

Our results indicate that experienced registered salespersons dealing with OTC drug sales were more likely to collect and provide information. Concerns about “interactions between OTC drugs and prescription drugs” were related to the frequency of information provision and information collection for proper medication use. The need for cooperation with pharmacists was generally high. A system that enables registered salespersons to consult with pharmacists, should be established.

## Supplementary Information

**ESM 1.**

## Data Availability

The datasets used and/or analyzed during the current study are available from the corresponding author on reasonable request. The data are not publicly available due to privacy or ethical restrictions.

## Abbreviations

OTC: over-the counter
