# Supplementary material for: Information provision and retrieval by registered salespersons from consumers during over-the-counter drug sales – a questionnaire survey
Source: BMC Health Serv Res. 2021 Dec 13;21:1333. doi: 10.1186/s12913-021-07343-x (PMC8667435; doi:10.1186/s12913-021-07343-x)
Supplement: Supplementary file 1 — ESM 1. [file 12913_2021_7343_MOESM1_ESM.docx]

**Supporting information**

Supplementary Table 1　Frequency of information provision and information collection by gender.

|  | Male（n=59） | Female（n=148） |
| --- | --- | --- |
| Information provision |  |  |
| Brand name | 48  （81.4） | 126  （85.1） |
| Indications | 58  （98.3） | 142  （95.9） |
| Dosage | 56  （94.9） | 137  （92.6） |
| Side effects * | 48  （81.3） | 95  （64.2） |
| Ingredients | 55  （93.2） | 132  （89.2） |
| Precautions for use | 43  （72.9） | 102  （68.9） |
| Precautions for storage and handling * | 30  （50.8） | 52  （35.1） |
| Information collection |  |  |
| Symptoms | 59  （100） | 146  （98.6） |
| Age | 47  （80.0） | 123  （83.1） |
| Medical history | 45  （76.3） | 110  （74.3） |
| Pregnancy (for consumers of relevant age and sex) * | 49  （83.1） | 102  （68.9） |
| Concomitant medications | 51  （86.4） | 132  （89.2） |
| Favorite items† | 27  （45.8） | 54  （36.5） |
| Allergies | 45  （76.3） | 112  （75.7） |

The number of subjects who answered “always” or “sometimes” was counted for each gender.

Numbers in () indicate percentage of all respondents.

* p<0.05

†Alcohol, tobacco, health foods, etc.

Supplementary Table 2　 Frequency of information provision and information collection by age group

|  | 20’s  （n=108） | 30’s  （n=60） | 40’s  （n=26） | 50’s  （n=8） | 60 or more  （n=5） |
| --- | --- | --- | --- | --- | --- |
| Information provision |  |  |  |  |  |
| Brand name | 93  （86.1） | 46  （76.7） | 23  （88.5） | 7  （87.5） | 5  （100） |
| Indications | 105  （97.2） | 57  （95.0） | 25  （96.2） | 8  （100） | 5  （100） |
| Dosage | 99  （91.7） | 56  （93.3） | 25  （96.2） | 8  （100） | 5  （100） |
| Side effects | 74  （68.5） | 39  （65.0） | 20  （76.9） | 7  （87.5） | 3  （60.0） |
| Ingredients | 97  （89.8） | 53  （88.3） | 24  （92.3） | 8  （100） | 5  （100） |
| Precautions for use | 77  （71.3） | 39  （65.0） | 21  （80.8） | 6  （75.0） | 2  （40.0） |
| Precautions for storage and handling | 42  （38.9） | 20  （33.3） | 12  （46.2） | 5  （62.5） | 3  （60.0） |
| Information collection |  |  |  |  |  |
| Symptoms | 107  （99.1） | 60  （100） | 25  （96.2） | 8  （100） | 5  （100） |
| Age | 84  （77.8） | 53  （88.3） | 21  （80.8） | 7  （87.5） | 5  （100） |
| Medical history | 74  （68.5） | 47  （78.3） | 21  （80.8） | 8  （100） | 5  （100） |
| Pregnancy (for consumers of relevant age and sex) | 81  （75.0） | 42  （70.0） | 19  （73.1） | 6  （75.0） | 3  （60.0） |
| Concomitant medications | 98  （90.7） | 50  （83.3） | 23  （88.5） | 7  （87.5） | 5  （100） |
| Favorite items† | 41  （38.0） | 23  （38.3） | 11  （42.3） | 4  （50.0） | 2  （40.0） |
| Allergies | 80  （74.1） | 44  （73.3） | 21  （80.8） | 7  （87.5） | 5  （100） |

The number of subjects who answered “always” or “sometimes” was counted for each age group. Because there were no subjects 10 years old or less, 10’s was excluded from the table. Numbers in () indicate percentage of all respondents.

* p<0.05

† Alcohol, tobacco, health foods, etc.

Supplementary Table 3　 Frequency of information provision and information collection by years of sales experience

|  | Less than 1 year  （n=32） | 1 - 3 years  （n=71） | 3 – 5 years  （n=29） | 5 years or more  （n=75） |
| --- | --- | --- | --- | --- |
| Information provision |  |  |  |  |
| Brand name | 28  （87.5） | 62  （87.3） | 25  （86.2） | 59  （78.7） |
| Indications | 30  （93.8） | 69  （97.2） | 29  （28.0） | 72  （96.0） |
| Dosage | 28  （87.5） | 66  （93.0） | 27  （93.1） | 72  （69.9） |
| Side effects | 19  （59.4） | 52  （73.2） | 17  （58.6） | 55  （73.3） |
| Ingredients | 25  （78.1） | 66  （93.0） | 27  （93.1） | 69  （92.0） |
| Precautions for use | 19  （59.3） | 56  （78.9） | 21  （72.4） | 49  （65.3） |
| Precautions for storage and handling | 9  （28.1） | 30  （42.2） | 13  （44.8） | 30  （40.0） |
| Information collection |  |  |  |  |
| Symptoms | 31  （96.9） | 70  （98.6） | 29  （100） | 75  （100） |
| Age | 23  （71.9） | 57  （80.3） | 25  （86.2） | 65  （86.7） |
| Medical history | 19  （59.4） | 51  （71.8） | 24  （82.8） | 61  （81.3） |
| Pregnancy (for consumers of relevant age and sex) | 19  （59.4） | 54  （76.1） | 24  （82.8） | 54  （72.0） |
| Concomitant medications | 26  （81.3） | 66  （93.0） | 28  （96.6） | 63  （84.0） |
| Favorite items† | 11  （34.4） | 28  （39.4） | 14  （48.3） | 28  （37.3） |
| Allergies | 20  （62.5） | 54  （76.1） | 24  （82.8） | 59  （78.7） |

The number of subjects who answered “always” or “sometimes” was counted in each category. Numbers in () indicate percentage of all respondents.

* p<0.05

†Alcohol, tobacco, health foods, etc.

Supplementary Table 4 Frequency of consultation with a pharmacist by registered salespersons when worries or anxieties arise about drugs

| N = 207 | Not at all | Not often | Neither | Usually | Always |
| --- | --- | --- | --- | --- | --- |
| Working with full-time pharmacist | 0  （0.0） | 3  （6.5） | 8  （17.4） | 21  （45.7） | 14  （30.4） |
| Not working with full-time pharmacist | 78  （48.4） | 36  （22.4） | 28  （17.4） | 13  （8.1） | 6  （3.7） |

Numbers in parentheses are percentages of all respondents who answered “yes” (upper row), or percentages of all respondents who answered “no” (lower row).
